# Regulation of PEST-containing nuclear proteins in cancer cells: implications for cancer biology and therapy

**DOI:** 10.3389/fonc.2025.1548886

**Published:** 2025-04-22

**Authors:** Kai-Chun Jiang, Yong-Hao Zhu, Zhi-Liang Jiang, Yi Liu, Wahab Hussain, Huang-Yin Luo, Wei-Hang Sun, Xin-Ying Ji, Ding-Xi Li

**Affiliations:** ^1^ Department of Traditional Chinese Medicine, Shu-Qing Medical College of Zhengzhou, Zhengzhou, Henan, China; ^2^ School of Stomatology, Henan University, Kaifeng, Henan, China; ^3^ Kaifeng Municipal Key Laboratory for Infection and Biosafety, Henan International Joint Laboratory of Nuclear Protein Regulation, School of Basic Medical Sciences, Henan University College of Medicine, Kaifeng, Henan, China; ^4^ Department of Urology, Institute of Urology, Sichuan University, Chengdu, China; ^5^ State Key Laboratory of Military Stomatology and National Clinical Research Center for Oral Diseases and Shaanxi International Joint Research Center for Oral Diseases, Center for Tissue Engineering, School of Stomatology, Fourth Military Medical University, Xi’an, Shaanxi, China; ^6^ Department of Oncology, Huaxian County Hospital, Anyang, Henan, China; ^7^ Faculty of Basic Medical Subjects, Shu-Qing Medical College of Zhengzhou, Zhengzhou, Henan, China; ^8^ The Affiliated Cancer Hospital, Zhengzhou University & Henan Cancer Hospital, Zhengzhou, China

**Keywords:** PCNP, PEST motif, ubiquitination, proteasome, cancer

## Abstract

The PEST-containing nuclear protein (PCNP) is a nuclear protein involved in the regulation of cell cycle progression, protein degradation, and tumorigenesis. PCNP contains a PEST sequence, a polypeptide structural motif rich in proline (P), glutamic acid (E), serine (S), and threonine (T), which serves as a proteolytic recognition signal. The degradation of specific proteins via the PEST sequence plays a crucial role in modulating signaling pathways that control cell growth, differentiation, apoptosis, and stress responses. PCNP is primarily degraded through the ubiquitin-proteasome system (UPS) and the calpain pathway, with phosphorylation of threonine and serine residues further accelerating its degradation. The ubiquitination of PCNP by the ring finger protein NIRF in an E3 ligase-dependent manner is well documented, along with its involvement in the MAPK and PI3K/AKT/mTOR signaling pathways. Additionally, PCNP is implicated in p53-mediated cell cycle arrest and apoptosis, which are essential for inhibiting tumor growth. To explore the role of PCNP in cancer, this review examines its effects on cell growth, differentiation, proliferation, and apoptosis in lung adenocarcinoma, thyroid cancer, ovarian cancer, and other malignancies derived from glandular epithelial cells. By focusing on PCNP and its regulatory mechanisms, this study provides a scientific basis for further research on the biological functions of the PEST sequence in tumor development and cancer progression.

## Graphical Abstract

A schematic representation illustrating how PEST-containing nuclear proteins are regulated in cancer cells. The PEST domain mediates key post-translational modifications (PTMs) that control protein stability, intracellular localization, and functional interactions with pivotal factors such as p53. These regulatory events influence critical cellular processes, including immune responses, metabolism, and transcription, ultimately shaping tumor progression. Targeting the stability, activation/inhibition, and localization of PEST NPs may provide novel strategies for cancer diagnosis and therapy (By Figdraw.).

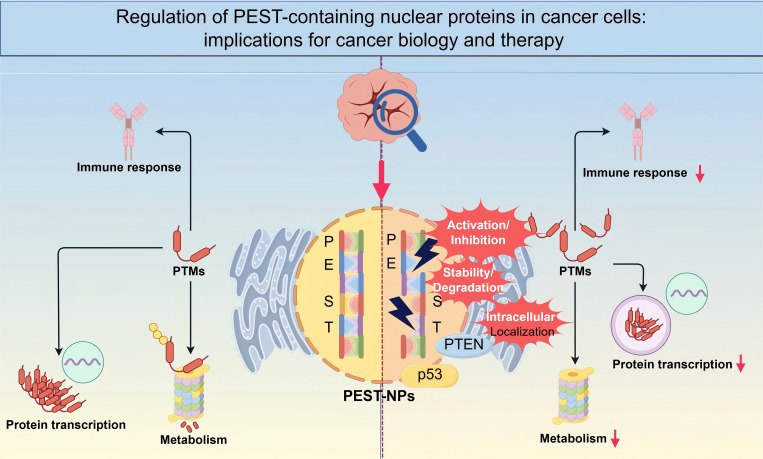

## 1 Introduction

A nuclear protein (NP) is a protein localized in the cell nucleus that acts as a barrier between the cytoplasm and nuclear membrane. NPs are crucial for maintaining cell cycle continuity by regulating key innate pathways. They play essential roles in controlling cell division, stem cell production, genomic stability, nuclear metabolism, and the structural integrity of cellular machinery, often through ubiquitination, an enzymatic post-translational modification that attaches ubiquitin to substrate proteins.

PCNP is a novel nuclear protein characterized by the presence of PEST sequence and short motifs composed of P, E, S, and T residues (1). Proteins such as kinases, phosphatases, transcription factors, and cyclins commonly contain PEST sequences (2). PCNP is a small, short-lived nucleoprotein comprising 178 amino acids and containing two prominent PEST sequences. Proteins with PEST sequences are involved in various physiological processes, including cell metabolism, nuclear-cytoplasmic transport, cell cycle regulation, signaling pathways, and nuclear protein stability. These sequences often serve as proteolytic recognition signals for targeted protein degradation and are considered determinants of protein instability due to their frequent association with unstable proteins (3, 4).

Hydrophilic domains that are at least 12 amino acids in length are commonly found in target proteins that degrade rapidly. PCNP has been shown to regulate protein ubiquitination and proteasomal degradation, thereby influencing protein stability and turnover (5). The “PEST hypothesis,” as reported in early studies, suggests that casein protein containing multiple PEST sequences are rapidly degraded in eukaryotic cells (6, 7). It is very important for PEST sequence degradation to turn on and off regulatory proteins that control cell growth, differentiation, the stress response, and programmed cell death (8). The PEST sequence is an intrinsically disordered region within certain protein sequences. This region can act as a phosphorylation site that recruits F-box-containing ubiquitin E3 ligases, facilitating ubiquitination and subsequent proteasomal degradation (9).

PCNP is capable of interacting with various cellular regulators, including promoters (cyclin E and cyclin D), tumor suppressors (p53, pRB, and PTEN), and cell cycle regulatory proteins, thereby influencing processes such as cell proliferation and apoptosis (10). Numerous studies have reported correlations between PCNP expression and the initiation and progression of several malignancies, such as neuroblastoma, lung adenocarcinoma, and ovarian cancer. PCNP may serve as a nuclear protein involved in tumor growth regulation, a transcriptional modulator, and a key regulator of the cell cycle (1, 11).

## 2 The potential physiological functions of PCNP containing PEST motif

### 2.1 Potential physiological functions of novel nuclear protein PCNP

PCNP plays a significant role in tumor development by regulating cell growth and proliferation, as demonstrated in studies on rheumatoid arthritis, pancreatic cancer, and leukemia (12). Multiple malignancies have implicated it in its broad expression across tissues.

PCNP was discovered through yeast two-hybrid screening investigations. It co-localizes with the Np95/ICBP90-like RING finger protein (NIRF) uniformly throughout the perinuclear area (13). NIRF has catalytic activity, and its N-terminus contains a ubiquitination domain, while its C-terminus has a ring finger catalytic domain. NIRF ubiquitinates PCNP as an E3 ligase, which controls its stability and possible role (14, 15).

PCNP works with NIRF. When NIRF is ubiquitinated, factors that help stop the cell cycle can also be ubiquitinated. These include cyclin E1, cyclin D1, and E3 ligase. Therefore, PCNP may be associated with NIRF in cell cycle regulation. NIRF controls phosphorylation and ubiquitination and is a key post-translational modifications (PTMs) in activating chromatin transcription. These facts show that PCNP ubiquitination is a key part of the chromatin-mediated transcription pathway modification complex (16, 17). Therefore, PCNP may be associated with NIRF in cell cycle regulation (18).

As a NIRF substrate, PCNP may go through histone PTM regulation like p53, which can change the activation of transcription factors and the expression of genes that follow. The ubiquitination-proteasome pathway (19) controls the breakdown of proteins through these transcription factors, which have short half-lives and PEST motifs.

PCNP interacts with several proteins, including E3 ubiquitin-protein ligase MARCH7, BMI1, TRAM1, and UHRF2. Overexpression of PCNP stops cancer cells from growing, migrating, and invading by encouraging apoptosis. However, its other role in tumor biology is still not fully understood. While PCNP shows promise as a therapeutic target, more research is needed to fully understand how it works to stop tumors from growing (20).

### 2.2 Physiological functions of the PEST motif and NPs

The PEST domain in protein tyrosine phosphatase PTPN18 has been shown to promote k48-linked HER2 ubiquitination through the proteasome pathway and regulate the negative feedback loop of the Human Epidermal Growth Factor Receptor EGFR-related gene 2 (HER2) (21). Similarly, studies indicate that deleting the PEST motif at the carboxyl-terminal of neuronal proliferation-differentiation control protein-1 (NPDC-1) enhances its stability and strengthens its inhibition of retinoic acid-mediated transcription (22). Additionally, removing the PEST sequence from the N-terminus of the homeobox transcription factor NANOG reduces its ubiquitination and improves its stability (23).

IjBNS, a nuclear IjB protein involved in thymic T-cell receptor activation, contains a PEST motif that regulates the polyubiquitination and rapid degradation of short-lived protein HAX-1, enabling swift cellular responses to various stimuli (24). Interestingly, the stability of the mouse IjBNS (mIjBNS) short-lived protein is governed by the proteasome rather than by the ubiquitination pathway (25).

The three lysine acyl residues in the PEST sequence are potential ubiquitin receptor sites and serve as attachment points for ubiquitin in proteins such as the yeast A-factor receptor (Ste3p) (26). For instance, oxidative stress in human neuroblastoma cells affects dysbindin-1A protein levels through phosphorylation of the PEST domain, marking it for proteasome degradation (27).

In summary, proteins containing PEST motifs primarily participate in protein degradation processes essential for cell growth, differentiation, stress responses, signaling pathway activation, and protein stability maintenance. The PEST domain serves as a critical recognition signal for protein hydrolysis (1, 23). The PEST motif mediates substrate degradation through mechanisms such as proteasomal breakdown, endocytosis of the yeast alpha-factor receptor (STE3), and lysosomal degradation of human calcium receptors (7, 28–33). Some of the things that NPs with PEST sequences do inside cells are nuclear transport, cell cycle control, nucleotide signaling, metabolism, immune response, DNA damage repair, and apoptosis (7).

NPs are involved in tumor growth or inhibition through post-translational modifications such as phosphorylation and ubiquitination. The two most crucial tumor suppressor proteins are p53 and PTEN (34). p53 is composed of 393 amino acids and can be triggered by factors like DNA damage, hypoxia, and mutations in other oncogenes. The type of post-translational modification determines the cell regulatory function associated with p53 (35). Also, p53 regulates cytoplasmic and nucleoplasmic reactions and determines the transcription and carcinogenicity of p53 by interacting with proteins (36).

Tumor suppressor protein PTEN is a nuclear protein containing two PEST sequences found in cancer cells in the form of mutations (37, 38). The middle part of the protein Myc consists of a PEST sequence responsible for ubiquitination, two conserved Myc boxes (BMI and MBIV), and an NLS responsible for nuclear aggregation (39). Myc is a resident nuclear transcription factor, which forms a dimer with MAX and affects cell growth and proliferation (40). The PEST region of PTEN is responsible for proteasome degradation through ubiquitination, a process that contributes to protein stability and suppresses tumor activity (41).

Acidic and hydroxylated residues in PEST motifs facilitate their ubiquitination, where lysine residues serve as ubiquitin acceptor sites, and proline residues influence polyubiquitin chain formation. The PEST motif’s size and charge have a direct effect on how well it ubiquitinates proteins, highlighting its role in localizing proteins within cells and triggering apoptosis by tumor suppressor proteins (1, 42).

## 3 The degradation process of nuclear protein PCNP

### 3.1 Ubiquitin-proteasome-mediated protein degradation pathway

The interaction between PCNP, a protein-specific substrate, and the ubiquitin ligase NIRF forms a novel cell signaling pathway that regulates the cell cycle and proliferation. NIRF contains several functional regions, including the YDG/SRA domain, PHD domain, and RING finger domain (14). As a ubiquitin ligase, NIRF binds to PCNP and changes how well ubiquitination works. This changes important cellular processes such as proliferation, apoptosis, migration, and invasion (15).

Identifying degradation signals in proteins is challenging due to the vast diversity of cellular proteins. Animal proteins broken down by proteasomes often have similar patterns. For example, the “-Asp-Ser-Gly-X-X-X-Ser-” sequence is found in human IκBα and β-catenin, and the “-Arg-Leu-Gly-X-X-X-Ile-Gly-” sequence is found in cyclins A, B1, and B2. These motifs also serve as phosphorylation sites, regulating protein stability (43–46).

In yeast, at least ten protein degradation signal motifs, including N-terminal degradation signals (N-degrons) and PEST sequences, have been identified (3, 47). PEST motifs are very important for controlling proteins after they have been made. They do this by preventing ubiquitination and proteasome degradation and by phosphorylating serine and tyrosine residues. This dual mechanism ensures precise protein expression control and maintains physiological function (48).

The ubiquitination system controls the breakdown of proteins (49). The system consists of three main enzymes: ubiquitin-activating enzyme E1, ubiquitin-conjugating enzyme E2, and ubiquitin ligase E3 (50). E1 activates ubiquitin by forming a thioester bond with its glycine residue, transferring it to E2. After that, E3 makes it easier for ubiquitin to attach for ubiquitin to attach to the lysine residue of the target protein, which tells proteasomes to break it down (51).

There aren’t many E2 enzymes in the human genome, but there are a lot of different E3 ligases that can bind to a lot of different substrates (52). A single connected ubiquitin residue is not enough to cause substrate degradation. For living cells to make a polyubiquitin chain (polyUb), they need to add a series of ubiquitin residues to the lysine residue that comes before it (53–55). Cell activity regulates this process. You can help the proteasome find the broken-down protein substrate by connecting a polyubiquitin chain to it. This chain is also one of the links that control protein degradation (56, 57).

### 3.2 Ubiquitination of PCNP

**Figure 1:**
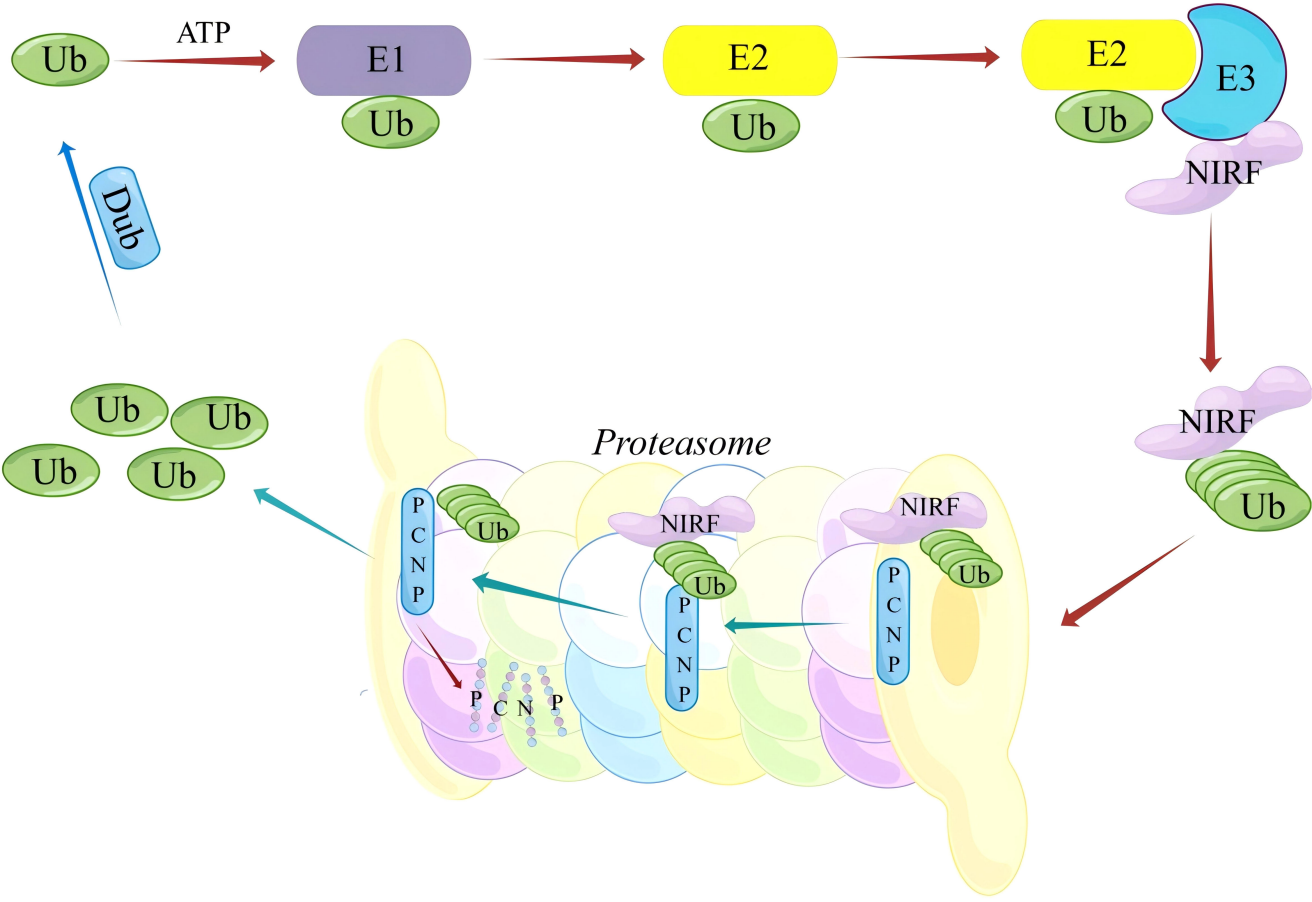
Schematic Representation of the NIRF-Mediated ubiquitin-dependent degradation pathway of PCNP.

Ubiquitination is a fundamental process that regulates protein stability, activity, and cellular localization. PCNP, characterized by its PEST sequences, is particularly susceptible to ubiquitination. Scientists have discovered that PCNP works with UHRF2 (also known as NIRF), a ubiquitin ligase that helps with DNA repair, cell cycle control via p53, and DNA methylation (1, 11) (**Figure 1**).

In the presence of ATP, the ubiquitin-activating enzyme (E1) initially binds free ubiquitin (Ub) and activates it, followed by the transfer of activated Ub to the ubiquitin-conjugating enzyme (E2). The E3 ubiquitin ligase NIRF subsequently recognizes and binds its substrate protein PCNP, catalyzing the covalent attachment of Ub to PCNP, thereby labeling PCNP for ubiquitination. Polyubiquitinated PCNP is then recognized and degraded by the 26S proteasome, completing the selective degradation of the substrate protein. This process is critical for maintaining intracellular protein homeostasis and regulating various cellular signaling pathways.

Experiments, such as co-immunoprecipitation and Western blotting, show that NIRF ubiquitinates PCNP. This suggests that NIRF plays a key role in changing how PCNP works in cancer biology. This interaction shows how important NIRF is as a target for studying how PCNP helps keep the genome stable and manages the cell cycle (58, 59). The ubiquitin-proteasome pathway breaks down many nuclear receptors, transcription factors, and oncogenes, including proteins such as cyclin D1 and E2F-1 (60), which are needed for proliferation and differentiation. Ubiquitination and deubiquitination maintain cellular homeostasis by dynamically regulating these processes (61).

Phosphorylation of PEST sequences is often used to control proteasome-mediated rapid protein turnover (62). The HPV E2 protein is involved in episomal genome maintenance, DNA replication, and gene transcription (63, 64). Phosphorylation at the flexible region speeds up the degradation of the PEST sequence. Edge stability around the PEST region can be adjusted by phosphorylation. PEST motif serine and tyrosine phosphorylation controls the stability and down-regulation of vascular endothelial growth factor receptor 2 (65). Reversible phosphorylation controls the biological function of many PEST-containing proteins, but it is not a crucial component of protein hydrolysis because the proteasome breaks down both phosphorylated and unphosphorylated proteins at the same rate (66). However, phosphorylation is not a crucial component of protein hydrolysis because the proteasome breaks down both phosphorylated and unphosphorylated proteins at the same rate. Point mutations can have stable or unstable effects, and phosphorylation, particularly on serine 301, disrupts the structure as a whole (67). There is a strong link between the half-life of the E2 protein with the same mutation *in vivo* and the thermodynamic stability of distinct peptides. Removing the PEST sequence significantly increased nuclear protein-related cell signaling receptor-mediated transcriptional inhibition. The normal cellular function of nuclear proteins and their potential role as tumor suppressors are regulated at the protein stability level (63). Therefore, conformational stability, rather than phosphate-modified recognition, regulates the degradation of the PEST sequence through a proteasome mechanism.

The PEST motif contains an abnormally high density of proline, glutamic acid, aspartic acid residues, hydroxylated amino acid serine, and threonine. Due to its PEST sequence, PCNP is inclined to ubiquitination mechanism in cells. NIRF is a potential ubiquitin ligase that can ubiquitinate PCNP, and proteins containing PEST are easily ubiquitinated (14). The overexpression of cytokines causes the activation of transcription factors controlled by ubiquitination. Nuclear proteins regulate transcription factors and cause the unrestricted development and spread of all human malignancies. In several signaling pathways, ubiquitination controls the location and activity of crucial protein kinases and the amounts of protein substrate (68).

Using NIRF to determine PCNP’s potential participation in different cancers is essential since PCNP is a transitory new oncogene, and its ubiquitination is a novel target for chemotherapy. These discoveries offer new research paths, allowing researchers to examine the interaction between NIRF and PCNP in various signaling pathways crucial to cell cycle regulation and genomic stability in greater depth.

### 3.3 Calpain pathway

The PEST motif also mediates protein degradation via the calcium-dependent cysteine protease calpain (69). This motif is distributed at both the amino- and carboxyl-terminal ends of proteins and often acts as a multifunctional contact site for protein interactions (70).

Maintaining the balance of protein levels is essential for the body’s physiological state. Calpain is a calcium-dependent neutral cysteine protease that is present in animal cells. Calpain1 and calpain2 are heterodimers of a regulatory component with a molecular weight of 28 kD and an 80 kD catalytic subunit (71). The Calpain family is closely related to cell cycle regulation, gene expression regulation, some apoptotic pathways, and long-term enhancement effects. It is also related to tumor proliferation, differentiation, invasion, and metastasis (72, 73).

The Calpain family is rich in the PEST sequence residue region, which can bind to calmodulin and participate in apoptosis (74). By cleaving molecules associated with apoptosis, including Bcl-2, Bax, Bid, and p53, activated calpains can engage in the pathological process of apoptosis and directly activate caspase-3 and caspase-7. Maintaining the permeability of the mitochondrial membrane *in vivo* depends on the concentration of calcium. ROS are produced when the concentration of mitochondrial calcium rises; this releases Cyt C and triggers apoptosis (75, 76).

Ca^2+^ concentration is crucial for preserving the permeability of the mitochondrial membrane *in vivo*. An increase in the mitochondrial calcium concentration will produce ROS, which releases Cyt C and causes apoptosis (77). Ca^2+^ can maintain the stability of the endoplasmic reticulum and regulate a large number of enzymatic reactions. Regular changes can stimulate endoplasmic reticulum stress (ER stress), open the JNK pathway, and stimulate Bax activation (78). Ca^2+^ can also regulate calpain, calpain cleavage Bax N-terminal, produce pro-apoptotic fragments, and promote apoptosis. As a regulatory subunit of calpain, calpain small subunit 1 (calpain-s1, capn4) affects the cell cycle, apoptosis, tumor occurrence, and development (79).

Calpain can affect integrin activation and focal adhesion formation by hydrolyzing the Q433-Q434 site of talin and the K2493-K2494 site of the talin-R end (80–82), thus affecting cancer cell migration, metastasis, and adhesion. The hydrolysis process of the TRPM4 protein inhibited the metastasis of colorectal cancer by mediating FAK protein dissolution and inhibiting the PI3K/Akt/mTOR signaling pathway. The PEST domain plays a crucial role in EGF/calpain-mediated proteolysis, which leads to the quick elimination of cyclin G2 (83).

The hydrolysis process of the TRPM4 protein inhibited the metastasis of colorectal cancer by mediating FAK protein dissolution and inhibiting the PI3K/Akt/mTOR signaling pathway (84). EGF/calpain-mediated proteolysis, which is triggered by EGF and causes cyclin G2 to be rapidly removed, requires the PEST domain (85). Numerous investigations have revealed that PCNP has a dual role in tumor promotion and inhibition and is strongly associated with the development of malignancies. It is evident that the PEST domain is required for calpain, much like the known tumor suppressor gene p53. PCNP can break down proteins through calpain, yet the exact process is still unknown.

## 4 Several common cancers associated with PCNP

Lung cancer is the leading cause of cancer-related mortality worldwide, encompassing small-cell lung cancer, squamous cell carcinoma, large-cell carcinoma, and adenocarcinoma (86, 87). Despite advances in treatment, the 5-year overall survival rate for patients with advanced or metastatic lung cancer remains below 20% (88, 89). Among non-small-cell lung cancers (NSCLC), adenocarcinoma represents a major subtype, with its incidence and mortality rates continuing to rise annually. Immune checkpoint inhibitors that work on programmed cell death protein 1 (PD-1) and its ligand (PD-L1) have the potential to make things better (90, 91). However, their clinical benefits are limited to a small subset of patients. Therefore, identifying novel molecular targets and developing more effective therapeutic strategies are critical for improving prognosis (92).

Neuroblastoma is the most common solid cancer in infants (93), originating from immature nerve cells, typically in the adrenal glands or along the sympathetic nervous system (94). While low- and intermediate-risk NB can often be resolved with surgery and chemotherapy, high-risk cases remain challenging, with 5-year survival rates improving to only 50% following comprehensive treatments such as radiotherapy, immunotherapy, stem cell transplantation, and chemotherapy. Recurrence and metastasis further complicate management (95).

Ovarian cancer (96), primarily of epithelial origin, arises from the fallopian tubes or peritoneum and most commonly affects women over the age of 50. It remains the leading cause of mortality among gynecological malignancies, with its incidence and death rates steadily increasing (97–99). Standard treatments include cytoreductive surgery and platinum-based chemotherapy. However, the emergence of multidrug resistance limits therapeutic efficacy, resulting in a 5-year survival rate below 45%. Recurrence and metastasis continue to pose significant clinical challenges (100).

Thyroid cancer, the most prevalent malignant tumor of the head and neck, has shown a sharp increase in incidence, particularly among women (101–103). Originating from follicular or parafollicular thyroid epithelial cells, most thyroid cancers are well-differentiated, slow-growing, and associated with low mortality (104). Nonetheless, early cervical lymph node metastasis is common, and even after radical surgery, patients face a high risk of recurrence and distant metastasis, adversely impacting survival and quality of life. Identifying molecular markers for high-risk lesions is crucial for guiding treatment and predicting prognosis (105).

PCNP has been detected in the nuclei of cancer cells, with its expression levels significantly differing between tumor and adjacent non-tumor tissues. Research indicates that PCNP overexpression and downregulation are associated with cancer cell proliferation, migration, and invasion. These findings highlight PCNP as a potential biomarker for cancer diagnosis and prognosis. Furthermore, its role as a therapeutic target is gaining attention, with efforts underway to develop PCNP inhibitors for cancer treatment.

## 5 Physiological functions of PCNP and p53 in tumor biology

The interchange of PCNP and p53 involves complex tumor suppression mechanisms since both proteins suppress tumors using separate concurrent techniques. PCNP controls cell proliferation via protein breakdown, whereas p53 regulates DNA damage responses through cell cycle arrest, apoptosis, and cellular senescence (106, 107). Enhanced cancer therapy strategies result from an understanding of how these two proteins work together.

### 5.1 PCNP’s role in collaboration with p53

Several regulatory systems that cooperate with p53 to create a more robust tumor-suppression system are how PCNP functions (108). The eradication of cancer cells is the most notable example of the simultaneous action between these proteins. Apoptosis results from PCNP’s support of the breakdown of Mcl-1 and survivin anti-apoptotic proteins. Through the transcriptional activation of PUMA and NOXA, the pro-apoptotic mechanism of p53 inhibits the activity of Bcl-2 family proteins (107, 109, 110). Mutations in these proteins can interfere with the cooperative functions of p53 proteins. Missense mutations in the DNA-binding domain of p53 that result in loss of function affect genomic surveillance and apoptotic pathways. Gain-of-function mutations negatively impact PCNP expression levels, disrupting proteostasis and increasing drug resistance, especially in glioblastoma cells and colorectal cancer cells. (111, 112). In malignancies with p53 wild-type, PCNP’s proteasomal activity serves as a therapeutic advantage. These healthy cells work to eliminate dangerous β-catenin proteins, which trigger the p53-mediated apoptosis process and create new avenues for Butlin-3a medication treatment. One of p53’s regulatory functions is TIGAR-mediated metabolic reprogramming, which inhibits the glycolytic route and increases the pentose phosphate pathway (113). The active status of PCNP in the nucleus is directly altered by antioxidant processes that are regulated by SESN2. By maintaining components like glucose-6-phosphate dehydrogenase (G6PD), the PEST domain of PCNP helps avoid p53-mediated metabolic limitations. This pathway keeps redox-sensitive transcription factors safe from oxidizing conditions and allows NADPH production to proceed in hypoxic tumors (114–116).

### 5.2 Integration of p53’s role in cell cycle arrest and apoptosis

The precise nature of PCNP and p53 cooperative behavior remains unknown in scientific terms. Through MDM2 ubiquitination contests, scientific research must determine whether PCNP promotes the stability of p53 after translation or how p53 interacts with PCNP promoter regions to start expression. Using p53-wild-type breast cancer models, proteomic assays show that PCNP enhances the stability of the p53 protein by destroying MDM2 through ubiquitin. The results of immunoprecipitation tests support these conclusions (117).TP53-PCNP genetic relationships should be investigated by studies using CRISPR-Cas9 technology (118). One way to pinpoint the precise tumor regions where PCNP and its target genes are found is using spatial transcriptomics. Physicians can produce synthetic lethal effects against p53-wild-type tumors by combining antisense oligonucleotides that target PCNP with idasanutlin MDM2 blockers. According to published studies, medical options for activating p53-mutant tumors through proteostasis rebound triggered by PCNP are still possible (119–121). The protein-protein interaction between p53 and PCNP regulates tumor development via cellular death mechanisms and metabolic breakdowns, opening up the possibility of personalized treatments for cancer resistance. The p53-PCNP interaction system uses a dynamic pattern of protein communication to create a situational tumor evolutionary route that determines specific cancer therapy strategies (122).

## 6 Mechanistic roles of PCNP in tumorigenesis and cancer progression

### 6.1 Dual role of PCNP in tumor regulation

The small nuclear protein PCNP contains two conserved PEST sequences and localizes inside nucleoplasmic eukaryotic cell regions. Its involvement has been studied in multiple cancer types. PCNP can either promote or inhibit tumor growth, depending on the tumor type and its regulatory mechanisms. Investigations into the ubiquitination-proteasome degradation pathway and RNA transcription regulation by PCNP highlight its dual role. PTMs of PCNP and its impact on target gene transcription play a pivotal role in modulating tumor progression (35).

Several unresolved questions remain regarding PCNP’s tumor-suppressive properties, particularly related to its PEST motifs, ubiquitination-proteasome degradation, and their influence on cell cycle regulation, transcription, and apoptosis. Mechanisms based on the ubiquitination-proteasome pathway are likely to define PCNP’s role in cancer therapy. The overexpression of PCNP, driven by both endogenous and exogenous apoptotic factors, correlates with increased PCNP ubiquitination during tumor proliferation.

Further exploration is needed to elucidate PCNP’s mechanisms in the central nervous, respiratory, and integumentary systems, as well as its structure-function relationships, to advance future research.

### 6.2 PEST motifs and ubiquitination in PCNP function

The tumor microenvironment hosts various nuclear proteins with PEST motifs, which are closely associated with tumor development. Tumor suppressor genes such as p53 and PTEN contain PEST motifs (123–125), as do oncogenes such as Bim-1 and Myc. Proteins like Mecp2, PICT-1, and PCNP exhibit heterogeneous characteristics in tumor regulation (126, 127). PEST sequences influence apoptosis, migration, and cell adhesion by targeting proteins for degradation. This process is tightly regulated by cell metabolic enzymes, transcription factors, protein kinases, phosphatases, and cyclins (128). PCNP is a significant regulatory element in tumor initiation and progression. It functions through multiple pathways to control cell proliferation in cancers such as neuroblastoma, lung adenocarcinoma, ovarian cancer, and thyroid cancer. The ubiquitin-proteasome pathway is crucial for degrading proteins undergoing rapid turnover, with the PEST region being the primary target for ubiquitination at the carboxyl terminus (129). Extracellular signal-regulated kinase (ERK)-controlled phosphorylation also regulates the degradation of PEST motifs by proteasomes (130).

Research shows that PCNP expression is significantly elevated in malignant tumors, including endometrial, cervical, colorectal, lung, and gastric cancers. Cancer cells often exhibit higher nuclear and cytoplasmic PCNP levels than adjacent tissues. Overexpression of PCNP correlates with poorer prognosis and increased aggressiveness in breast cancer, positioning it as a potential biomarker and therapeutic target (131). For instance, in colorectal cancer, PCNP interacts with critical signaling pathways controlling cell division and apoptosis, with inhibition of these pathways limiting tumor growth (132). Similarly, PCNP impacts oncogenic pathways in lung cancer, where inhibiting its activity suppresses tumor progression, indicating a potential therapeutic approach (16). PCNP also influences androgen receptor activation in prostate cancer, making it a viable target for hormone-driven malignancies (133).

The role of PCNP in cancer biology varies among malignancies, reflecting its complexity. Understanding these specific pathways is critical for designing tailored therapies to improve patient outcomes. While progress has been made, further research is necessary to fully elucidate PCNP’s therapeutic potential across different cancers.

### 6.3 Lysosomal-mediated degradation of PCNP

PCNP breakdown is predominantly accomplished through UPS proteolysis and calpain-dependent proteolysis, while there is no conclusive evidence that lysosomal pathways play this role. The proline-glutamic acid-serine-threonine-rich PEST motifs in PCNP allow UHRF2 (NIRF), acting through ubiquitination, to target this protein for UPS degradation, a critical mechanism in cell cycle control and chromosomal preservation (134). Calpain, a calcium-dependent protease, regulates the cleavage of PEST motifs by neurotopic granule proteins, which influences apoptosis while controlling cell mobility (135). Lysosomal degradation of PCNP has not yet been proven, however researchers speculate on connection between distinct protein clearance mechanisms. Autophagy seems as a lysosome-dependent process that can take over damaged UPS functions based on observations of PEST-containing proteins including p53 and Myc when study subjects get proteasome inhibitor treatment (136, 137). There is evidence that PCNP may enter lysosomes by K63-linked ubiquitination, particularly under hypoxic and nutrient-depleted stress situations, albeit this possible pathway has not yet been rigorously validated (138, 139). The use of the lysosome inhibitor chloroquine may stabilize tumors in which PCNP acts as a tumor suppressor; however, the anti-tumor impact may be enhanced when coupled with the proteasome inhibitor bortezomib. Research limitations include the absence of observational evidence for lysosomal PCNP breakdown, an unproven association between PCNP and lysosomal indicators utilizing LAMP1, and experimental techniques for ubiquitin chain type characterization or lysosomal inhibition (1, 15). Future research should focus on these regulatory systems, particularly in malignancies that exhibit treatment resistance, in order to develop novel therapeutic approaches.

### 6.4 Autophagic regulation by PCNP in cancer

Autophagy control by PCNP has potential, but researchers have yet to completely investigate this process, which is still relevant in cancer biology studies. According to current research, the leukemic protein 57 imprint exhibits both UPS and calpain-mediated proteolytic capabilities, as well as putative autophagic pathway interaction, in stressful therapy-resistant situations. Stress processes regulate autophagy start via the PI3K/AKT/mTOR axis, and evidence suggests PCNP plays a function in these autophagy control systems. In hypoxic tumors, PCNP expression stabilizes HIF-1α (138) and inhibits the formation of autophagic structures by activating glycolytic processes through transcription. PCNP follows UPS breakdown pathways in nutrient-depleted conditions, allowing FOXO3 to trigger the autophagic elimination of damaged cell structures (139, 140). PCNP’s PEST motifs allow for control over protein stability, which influences significant autophagy-related proteins such as Beclin-1 and LC3, as well as transcription factors and cyclins (135), in ways that are similar to the regulation mechanisms of these protein classes. Ovarian cancer cells stimulate Wnt/β-catenin signaling through PCNP, producing survivin and other β-catenin/TCF4 target genes that prevent autophagic cell death (136, 137). Neuroblastoma cells exposed to chemotherapy use accelerated autophagy as a survival strategy due to PCNP-MYCN binding, demonstrating PCNP’s multiple impact on tumor formation.

The therapy of the PCNP-autophagy pathway has the potential to be a successful therapeutic method. Combining chloroquine prescription treatments with PCNP inhibitor medicines, such as antisense oligonucleotides, may result in increased apoptotic effects for cancer cells expressing high PCNP levels. Tumors with low PCNP should respond to treatment stabilization because it will disrupt their faulty autophagy pathways. Scientists should conduct experimental testing of these concepts using LC3-II flux assays coupled with PCNP-autophagy co-immunoprecipitation analysis of ATG5-ATG12 complexes and by comparing autophagy markers between PCNP-knockout and wild-type tumors in live people (62, 141).

### 6.5 PCNP-mediated chromatin-modulated transcription

PCNP regulates chromatin-mediated transcription through interactions with ubiquitination pathways, epigenetic modifiers, and transcriptional complexes, shaping oncogenic and tumor-suppressive gene networks. Central to its function is its partnership with the E3 ubiquitin ligase NIRF, which ubiquitinates PCNP, linking its stability to chromatin dynamics. In neuroblastoma, the PCNP-NIRF complex binds promoters of cell cycle genes, recruiting histone deacetylases (HDACs) and DNA methyltransferases (DNMTs) to repress transcription and enforce cell cycle arrest (136). Conversely, PCNP degradation under stress conditions relieves this repression, enabling oncogene activation. PCNP also modulates transcription factors via its PEST motifs, stabilizing β-catenin in ovarian cancer by degrading its ubiquitin ligase β-TrCP, which promotes β-catenin/TCF4-driven transcription of oncogenes like MYC and CCND1. In the p53 pathway, PCNP enhances p53 ubiquitination by MDM2 under basal conditions, but DNA damage disrupts this interaction, freeing p53 to activate pro-apoptotic genes (134). Epigenetically, PCNP recruits BRCA1 to ubiquitinate histone H2A at lysine 119, silencing polycomb-target genes and suppressing tumor stemness, while its interaction with DNMT3A facilitates hypermethylation of tumor suppressor promoters, silencing these genes in lung adenocarcinoma (136). Under hypoxia, ERK-phosphorylated PCNP dissociates from antioxidant gene promoters, enabling ROS mitigation, whereas in nutrient-deprived tumors, PCNP stabilizes HIF-1α by blocking VHL-mediated ubiquitination, driving glycolytic gene transcription. PCNP’s dual roles manifest in colorectal cancer, where it activates MYC and SNAI1 via β-catenin to promote epithelial-mesenchymal transition (EMT) and metastasis (140), and in glioblastoma, where it recruits HDAC1 to silence oncogenic enhancers like EGFRvIII. Therapeutically, HDAC inhibitors may counter PCNP-driven histone acetylation in glioblastoma, while small molecules enhancing PCNP-NIRF interaction could silence oncogenes in MYC-driven cancers. Future studies must map PCNP’s genome-wide binding sites, identify synthetic lethal partners via CRISPR screens, and test epigenetic therapies in PCNP-high tumors (62, 136).

### 6.6 PCNP-regulated signaling pathways in cancer

**Figure 2:**
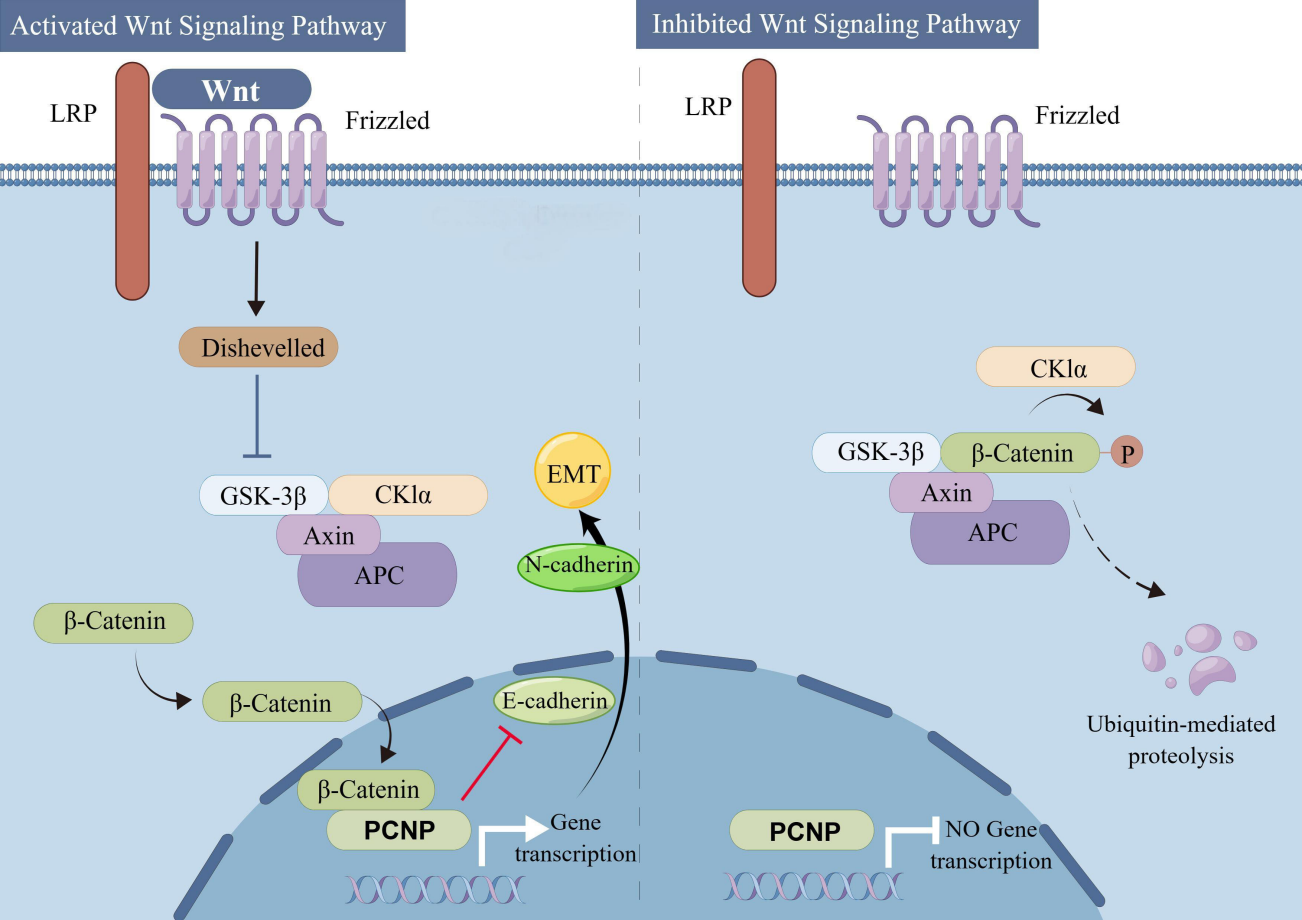
Activated and inhibited Wnt signaling pathways and their impact on β-catenin dependent gene transcription. A schematic overview comparing the activated (Left) and inhibited (Right) Wnt signaling pathways. In the activated pathway, Wnt ligands bind to Frizzled (Fzd) and low-density lipoprotein receptor-related protein (LRP) co-receptors, leading to Dishevelled (Dvl)-mediated inhibition of the β-catenin destruction complex (consisting of GSK-3β, CK1α, Axin, and APC). Stabilized β-catenin accumulates in the cytoplasm, translocates to the nucleus, and promotes transcription of target genes, including PCNP, thereby driving processes such as the epithelial-mesenchymal transition (EMT) via modulation of E-cadherin and N-cadherin expression. In the inhibited pathway, lack of Wnt ligand or the presence of inhibitory signals maintains the destruction complex in an active state, leading to phosphorylation and subsequent ubiquitin-mediated degradation of β-catenin. This prevents β-catenin from entering the nucleus and suppresses transcription of downstream target genes, including PCNP (By Figdraw.).

PCNP orchestrates a complex network of signaling pathways in cancer, modulating transcription, cell survival, and metastasis through interactions with ubiquitination systems, metabolic regulators, and stress-response mechanisms. Central to its function is its regulation by the UPS, where the E3 ligase NIRF ubiquitinates PCNP, targeting it for degradation to suppress proliferation in cancers like ovarian carcinoma, while NIRF inhibition stabilizes PCNP in glioblastoma to enhance tumor-suppressive activity. PCNP also stabilizes β-catenin by degrading its ubiquitin ligase β-TrCP, enabling β-catenin/TCF4-driven transcription of oncogenes and promoting EMT in colorectal and ovarian cancers (140). Under hypoxic stress, PCNP stabilizes HIF-1α by blocking VHL-mediated ubiquitination, driving glycolytic gene expression to support tumor survival, while ERK-dependent phosphorylation of PCNP activates PI3K/AKT/mTOR signaling, enhancing cell growth and inhibiting autophagy. In the p53 pathway, PCNP facilitates MDM2-mediated ubiquitination of p53 under normal conditions, but DNA damage disrupts this interaction, allowing p53 to activate pro-apoptotic genes and enforce cell cycle arrest via p21. PCNP further suppresses autophagy by stabilizing mTORC1, though proteasome inhibition can trigger compensatory lysosomal degradation, suggesting therapeutic synergy between autophagy inhibitors and proteasome blockers. The pathway interplay is illustrated as follows: hypoxia stabilizes HIF-1α via PCNP, driving glycolysis; NIRF ubiquitinates PCNP for proteasomal degradation, influencing cell survival; β-catenin stabilization by PCNP activates EMT/metastasis; and ERK-phosphorylated PCNP activates PI3K/AKT/mTOR to inhibit autophagy. Therapeutically, targeting these pathways—such as using Wnt inhibitors in β-catenin-driven tumors or HDAC inhibitors to counteract PCNP-mediated histone deacetylation—holds promise for precision oncology (62) (**Figure 2**).

## 7 Role of PCNP in cancer therapeutics

### 7.1 Therapeutic potential of PCNP in cancer treatment

Oncology research shows promise in targeting PCNP for therapy because it plays tumor suppressive and cancer-inducing roles toward malignancies. Clinical researchers aim to create targeted therapy by understanding the multiple effects of PCNP on ubiquitination-proteasome signaling pathways and signaling network control and by analyzing its variable expression patterns. Ubiquitin ligase NIRF inhibitor molecules enable the therapeutic destabilization of PCNP in cancer cells that exhibit solid growth characteristics including colorectal and ovarian malignancies (108, 117). PCNP functions as a tumor suppressor when treated with proteasome inhibitors such as bortezomib in cases where anaplastic thyroid cancer or glioblastoma have lost PCNP and become more unstable (120, 136).

Synthetic lethality provides one essential therapeutic approach in cancer treatment. Medical research indicates that simultaneous treatment of PCNP and MYC has potential to boost treatment outcomes within tumors that express high levels of MYC such as neuroblastoma and breast cancer (11, 108, 136). Laboratory experiments with ASOs targeting PCNP in neuroblastoma test tubes found the oligonucleotides decreased tumor growth alongside MYCN protein amounts thus demonstrating potential value in MYC-driven cancer treatment (136, 142). The combination of PCNP inhibition with PARP inhibitors could take advantage of DNA repair deficiencies in PCNP-deficient tumors as researchers do with BRCA-mutant cancers (11, 119).

The therapeutic importance of PCNP becomes even more substantial due to its contribution to chemoresistance development. The activation of Wnt/β-catenin signaling by PCNP in ovarian cancer cells induces platinum resistance and EMT transition which may be treated effectively through combined the use of Wnt inhibitors such as PRI-724 and EMT-targeted drugs including FAK inhibitors (115). The regulatory role of PCNP over MAPK and PI3K/AKT/mTOR cellular pathways makes it suitable for use as an indicator for drug response to kinase inhibitors. PCNP’s elevated presence in lung adenocarcinoma cells activates ERK signaling therefore indicating trametinib-based MEK inhibitor therapy responsiveness (120).

Immunotherapy applications are also emerging. Studies reveal that breast and colorectal cancers showing elevated PCNP expression levels develop suppressive immune environments that have high PD-L1 activity and exhausted T-cells (119). The reduction of PCNP expression creates a more visible tumor to the immune system through MHC-I expression enhancement which could indicate successful treatment when combined with PD-1/PD-L1 blocking drugs (119). Research shows that PCNP acts as a valuable target for treating enzalutamide resistance in prostate cancer by activating the androgen receptor because early-phase trials have started using PCNP ASOs to treat AR-positive metastatic disease (62, 136).

### 7.2 Development of small molecules/drugs targeting PCNP

The dual effects of PCNP on cancer development require innovative research targeting this molecule through small molecules or biologic approaches. One method works by controlling ubiquitination processes because the E3 ligase NIRF controls PCNP stability by marking it for proteasomal elimination (1). Research molecules that activate NIRF interaction with PCNP to break down oncogenic PCNP in colorectal and ovarian cancers but strive to design RING domain inhibitors that protect PCNP in tumors like glioblastoma. Besides targeting PCNP’s PEST motifs through chemical compounds either by creating peptidomimetics that enhance stability or disrupting its binding to ubiquitin ligase when PEST motifs are overexpressed in cancer cells. Selective PCNP degradation through PROTACs allows the connection of PCNP-binding peptides to E3 ligase recruiters that force ubiquitination of the target protein (15). Allosteric inhibitors developed from structural knowledge of PCNP could stop its binding to oncogenic partners including β-catenin and MYCN (143). The continued advancement of PCNP-targeted therapies encounters two main hurdles because PCNP shows tissue-specific roles requiring precise targeting while patients require biomarkers to separate groups based on PCNP amplification or PEST mutation status. The development of drugs for neuroblastoma or glioblastoma becomes more challenging because of barriers posed by the blood-brain barrier thus forcing studies to use nanoparticles and small molecules for treatment delivery (137). Initial developments in NIRF inhibition research lead to the development of half-methylated DNA mimetic structures that destabilize PCNP in prostate cancer tissues while antisense oligonucleotides successfully reduce PCNP mRNA levels in MYCN-driven neuroblastoma cases (11, 142). The drug bortezomib represents one example of protease inhibitor use for PCNP stabilization even though it lacks target-specificity thus prompting researchers to develop new compounds. The future plans will focus on drug combinations between PCNP-specific medications connected to PARP inhibitors and kinase inhibitors combined with nano-size delivery technology and AI-driven drug development to maximize drug performance and resistance avoidance.

Despite this promise, challenges remain. The tissue-specific characteristics of PCNP require precise targeting strategies so that toxic effects get minimized especially in normal tissues which depend on essential biological operations including DNA repair. Nanoparticles loaded with siRNA liposomes or CRISPR tools show promise to improve the specificity of anti-PCNP treatment (144) The translation of biomarker-driven patient stratification for clinical purposes depends on evaluating PCNP expression levels and PEST motif mutations together with ubiquitination status as diagnosis criteria (145).

## 8 Limitations in current research on PCNP

Numerous studies have highlighted the role of PCNP PTMs in regulating transcriptional activation or repression of target genes, influencing various cellular responses. Despite PCNP’s unique features, including its PEST motif and involvement in the ubiquitination-proteasome degradation pathway, significant gaps remain in understanding its role in tumor suppression (146, 147). These gaps are particularly evident in processes related to cell cycle regulation, transcription, and apoptosis (148).

The dual behavior of PCNP overexpression, modulated by endogenous and exogenous apoptotic execution factors, is associated with increased ubiquitination during tumor proliferation (149). This characteristic, grounded in the ubiquitination-proteasome pathway, positions PCNP as a promising therapeutic target for cancer treatment. However, additional research is needed to elucidate the underlying mechanisms of PCNP across various diseases, including disorders of the central nervous, respiratory, and integumentary systems. Enhancing our understanding of PCNP’s structure and function will facilitate future investigations.

Recent advances in genomic and proteomic technologies have deepened our understanding of PCNP’s role in cancer biology. For instance, studies in breast cancer have uncovered molecular pathways linking PCNP to tumorigenesis (150). CRISPR-Cas9 gene-editing techniques have been employed in colorectal cancer research to precisely knock out PCNP, revealing its impact on tumor growth and metastasis (151). In lung cancer studies, patient-derived xenograft models are increasingly used to mimic the tumor microenvironment and validate PCNP as a therapeutic target (152). Additionally, novel PCNP inhibitors are under investigation in prostate cancer trials, with encouraging preclinical results (153).

To provide a comprehensive assessment of the current state of PCNP research, it is essential to acknowledge existing limitations and potential biases. Incorporating the latest findings and critically analyzing these data will enhance the scientific credibility of future studies, enabling a more nuanced understanding of PCNP’s role in cancer biology and guiding subsequent research directions.

Currently, PCNP is recognized as a pivotal regulatory protein in carcinogenesis, playing a critical role in cell cycle regulation. Its ability to mediate apoptosis and cell proliferation mechanisms underscores its potential as a molecular target for cancer therapies. As research progresses, PCNP holds significant promise as a foundation for innovative therapeutic strategies aimed at managing tumor growth and progression.

## 9 Concluding remarks

PCNP, a recently identified oncogene, is a short-lived nucleoprotein containing PEST sequences. Its regulation through ubiquitination and proteasome degradation has emerged as a potential target for chemotherapy. Previous studies have shown that PCNP influences cancer cell growth, the cell cycle, and autophagy through diverse pathways.

Despite these findings, the molecular mechanisms underlying PCNP’s role in tumorigenesis remain poorly understood, leaving several key questions unanswered. What is the precise biological function of PCNP as a nuclear protein? How does it contribute to protein degradation? What are the roles of PCNP-associated macromolecules? Addressing these questions is critical to fully elucidate PCNP’s function in cancer biology.

The concentration gradients of PCNP within and outside the nucleus significantly impact cellular processes such as proliferation, apoptosis, migration, invasion, and tumorigenesis. Unique characteristics of PCNP, including its PEST motifs, ubiquitination, and proteasome-mediated degradation, link it to cell cycle regulation. Understanding the molecular mechanisms by which PCNP exerts its effects in cancer cells is vital for developing effective diagnostic, therapeutic, and prognostic strategies for cancer.

Resolving the ambiguities surrounding PCNP’s role in tumor development and its potential as a therapeutic target can pave the way for novel cancer treatments. Advancing research into PCNP’s structure and function will deepen our understanding of its role in tumorigenesis, facilitating the translation of PCNP-based drugs from preclinical studies to clinical applications. Additionally, investigating other nucleoproteins containing PEST motifs represents a promising avenue for advancing cancer diagnosis and treatment.

Given the critical influence of PCNP on cell proliferation and apoptosis, further elucidation of its underlying mechanisms is essential. This knowledge will contribute to the design of effective PCNP-based diagnostic and therapeutic strategies, accelerating their application in clinical oncology.

## Glossary

PCNP: PEST-containing nuclear protein
P: proline
E: glutamic acid
S: serine
T: threonine
UPS: ubiquitin-proteasome-mediated protein degradation pathway
NP: Nuclear protein
NIRF: Np95/ICBP90-like RING finger protein
PTM: post-translational modification
HER2: human epidermal growth factor receptor EGFR-related gene 2
NIRF: Np95/ICBP90-like RING finger protein
NPDC-1: neuronal proliferation-differentiation control protein-1
NSCLC: non-small-cell lung cancers
PD-1: programmed cell death protein 1
NB: Neuroblastoma
G6PD: glucose-6-phosphate dehydrogenase
ERK: extracellular signal-regulated kinase
HDACs: histone deacetylases
DNMTs: DNA methyltransferases
EMT: Epithelial-mesenchymal Transition.
